# Neddylation Regulation of Immune Responses

**DOI:** 10.34133/research.0283

**Published:** 2023-12-07

**Authors:** Hongmei Mao, Xin Lin, Yi Sun

**Affiliations:** ^1^ Cancer Institute (Key Laboratory of Cancer Prevention and Intervention, China National Ministry of Education) of the Second Affiliated Hospital and Institute of Translational Medicine, Zhejiang University School of Medicine, Hangzhou 310029, China.; ^2^ Institute for Immunology, School of Medicine, Tsinghua University, Beijing 100084, China.; ^3^ Changping Laboratory, Beijing 102206, China.; ^4^ Cancer Center of Zhejiang University, Hangzhou 310029, China.; ^5^ Zhejiang Provincial Clinical Research Center for Cancer, Hangzhou, Zhejiang Province, China.; ^6^ Key Laboratory of Molecular Biology in Medical Sciences, Hangzhou, Zhejiang Province, China.; ^7^ Research Center for Life Science and Human Health, Binjiang Institute of Zhejiang University, Hangzhou 310053, China.

## Abstract

Neddylation plays a vital role in post-translational modification, intricately shaping the regulation of diverse biological processes, including those related to cellular immune responses. In fact, neddylation exerts control over both innate and adaptive immune systems via various mechanisms. Specifically, neddylation influences the function and survival of innate immune cells, activation of pattern recognition receptors and GMP-AMP synthase–stimulator of interferon genes pathways, as well as the release of various cytokines in innate immune reactions. Moreover, neddylation also governs the function and survival of antigen-presenting cells, which are crucial for initiating adaptive immune reactions. In addition, neddylation regulates T cell activation, proliferation, differentiation, survival, and their effector functions, thereby ensuring an appropriate adaptive immune response. In this review, we summarize the most recent findings in these aspects and delve into the connection between dysregulated neddylation events and immunological disorders, especially inflammatory diseases. Lastly, we propose future directions and potential treatments for these diseases by targeting neddylation.

## Introduction

### Neddylation modification

**Fig. 1.**
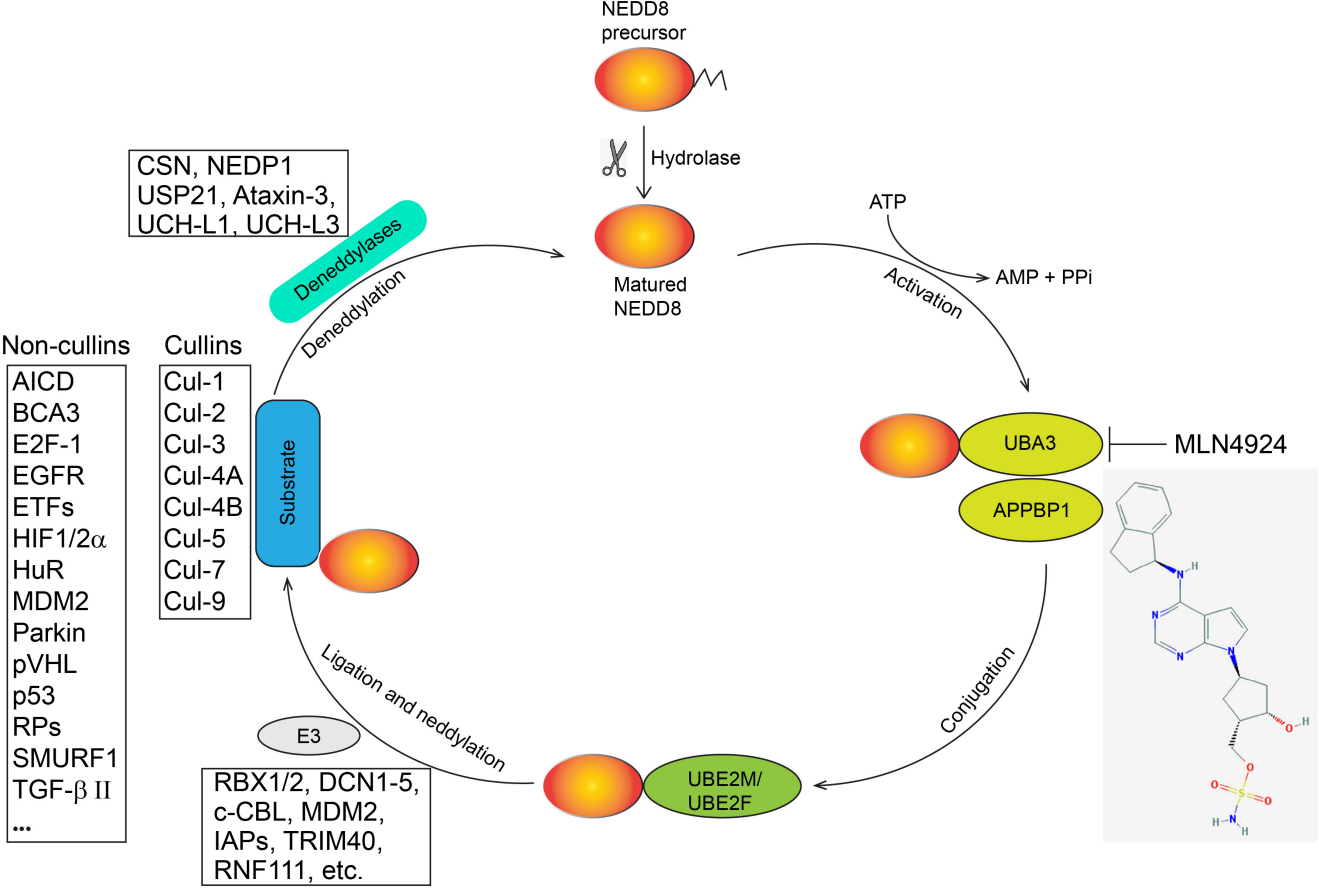
Neddylation modification. The process of neddylation modification of a protein begins with the proteolytic cleavage of the NEDD8 precursor. Mature NEDD8 is covalently attached to a lysine residue on the substrate protein, catalyzed by the NEDD8-activating enzyme E1, the NEDD8-conjugating enzyme E2, and substrate-specific NEDD8 E3 ligases. The removal of the NEDD8 molecule is catalyzed by deneddylases.

Protein neddylation is a biochemical process in which a ubiquitin-like molecule NEDD8 (neuronal precursor cell-expressed developmentally down-regulated protein 8) is attached to a lysine residue within a substrate protein [1] (Fig. 1). The neddylation process initiates with NEDD8 maturation, where the precursor undergoes proteolytic processing, exposing a C-terminal Gly residue [2]. The mature NEDD8 undergoes adenosine triphosphate (ATP)-dependent activation facilitated by the NEDD8-activating E1 enzyme (NAE) [3]. Following activation, NEDD8 is trans-thiolationed to an NEDD8-conjugating E2 enzyme [4]. The final stage involves covalent attachment of NEDD8 to a lysine residue within the substrate protein, catalyzed by a substrate-specific E3 ligase.

In mammalian cells, there exists a singular E1 heterodimer, comprising the regulatory subunit amyloid-β precursor protein-binding protein 1 and the catalytic subunit ubiquitin-activating enzyme 3 (UBA3) [3]; 2 E2 enzymes, UBE2M (also known as UBC12) or UBE2F [4]; and a dozen of E3 ligases, such as RBX1 (also known as ROC1), RBX2 (also known as ROC2, SAG, and RNF7), DCN1 to DCN5, inhibitors of apoptosis, murine double minute 2, casitas B-lineage lymphoma, ring finger protein 111 (RNF111), and tripartite motif-containing 40, among others [5]. Notably, all these E3 ligases also serve as E3s for ubiquitylation.

Unlike ubiquitylation, neddylation of a specific protein does not result in its degradation by the proteasome; instead, it influences its stability, conformation, subcellular localization, and function [6]. The main targets of neddylation are the cullin subunits of Cullin-RING ligases (CRLs), including Cul-1, Cul-2, Cul-3, Cul-4A, Cul-4B, Cul-5, Cul-7, and Cul-9. Neddylation induces conformational changes in cullins, leading to activation of CRLs, the largest family of E3 ubiquitin ligases, which, in turn, promote the ubiquitylation and degradation of numerous short-lived signaling proteins. This process is pivotal in controlling various biochemical and biological processes, including signal transduction, cell cycle progression, DNA replication and repair, gene transcription, stress responsiveness, viral infection, and tumorigenesis, among others [7].

Neddylation represents a dynamic process that can be reversed through the removal of NEDD8 from a substrate protein, catalyzed by deneddylase, a process known as deneddylation [8]. Notable deneddylases include the COP9 signalosome (CSN), NEDP1 (also known as DEN1 or SENP8), USP21, Ataxin-3, UCH-L1, and UCH-L3. Of these enzymes, only NEDP1 and CSN act as NEDD8-specific enzymes, while the others also function as deubiquitylases [2]. CSN5, the primary form of cullin deneddylase, is a zinc metalloprotease composed of 8 subunits [9], whereas NEDP1 operates as a cysteine protease with a specific affinity for NEDD8, enabling it to selectively detach NEDD8 from a neddylated substrate. Mutating the cysteine residue to alanine abolishes its protease activity [10].

MLN4924, also designated as pevonedistat, is a robust and highly selective small-molecule inhibitor of UBA3, the catalytic unit of neddylation E1, thus inhibiting the entire process of neddylation modification. Currently, it is being evaluated in numerous Phase I/II/III clinical trials to assess its potential as an anticancer therapy. These trials explore its efficacy both when used as a standalone treatment and in combination with chemotherapy [11]. Structural analysis has revealed that MLN4924 forms an adduct between the C-terminus of NEDD8 and the ATP-binding site of UBA3, effectively blocking further enzymatic processes and resulting in the deactivation of CRLs [12]. As a result of this inhibition, various CRL substrates accumulate, thereby regulating numerous cellular processes. Many preclinical studies have shown that when administered as a standalone treatment, MLN4924 effectively restrains various types of cancer cells by inducing growth arrest, apoptosis, autophagy, and senescence. Furthermore, when incorporated into combination therapies, it enhances the sensitivity of cancer cells to chemotherapy and radiation [13]. Apart from its direct impact on cancer cells, MLN4924 also exerts effects on the tumor microenvironment (TME). It accomplishes this by modulating the activities of immune cells such as macrophages, dendritic cells (DCs), and T cells, as well as cancer-associated fibroblasts (CAFs), among others [14–16]. Thus, MLN4924 shows promising potential as an anticancer agent by its ability to disrupt neddylation, then inactivate CRLs, and subsequently regulate various cellular and micro-environmental processes critical for cancer growth and progression.

### Immune responses

The immune response is a crucial biological process that protects the host from microbial infections. The initial defense mechanism comprises the body’s physical and chemical safeguards, such as the skin, mucus, cilia, tears, stomach acid, and urinary flow [17]. However, when pathogens manage to evade these barriers, the secondary nonspecific defense, known as the innate immune response, is activated. The innate immune system initiates various protective mechanisms, including inflammation, pathogen engulfment, and the release of proteins/cytokines [18,19]. These intricate processes recruit specialized defense cells due to the activation of signaling pathways involving pattern recognition receptors (PRRs) and the cyclic GMP-AMP synthase (cGAS)–stimulator of interferon genes (STING) pathway. Among the recruited defense cells are phagocytes and natural killer (NK) cells. The primary types of phagocytes include monocytes, neutrophils, eosinophils, tissue DCs, macrophages, and mast cells [20]. The PRRs present on phagocytes are capable of recognizing conserved molecular structures referred to as pathogen- or damage-associated molecular patterns (PAMPs and DAMPs), which are commonly encountered in microorganisms or damaged, injured, or stressed cells [21,22]. The PRRs are categorized into 4 distinct families: Toll-like receptors (TLRs), nucleotide-binding oligomerization domain-like receptors (NLRs), C-type lectin receptors (CLRs), and retinoic acid-inducible gene I (RIG-I)-like receptors (RLRs). Upon activation, these receptors trigger a range of cellular responses, including the initiation of signaling pathways such as the mitogen-activated protein kinase (MAPK) and nuclear factor-κB (NF-κB) pathways, leading to the production of inflammatory cytokines and chemokines. These molecules are pivotal in the elimination of pathogens [23]. Additionally, the cGAS receptor is responsible for detecting cytosolic microbial and tumor-derived DNA [24]. Activation of the cGAS-STING pathway triggers the de novo synthesis of antiviral type I interferons, a subclass of cytokines and related gene products that work together to clear pathogens [25].

Another class of cytokines is interleukin (IL) with a primary function in the regulation of the activation, differentiation, proliferation, maturation, migration, and adhesion of both innate and adaptive immune cells [26]. In cases where pathogens manage to bypass the innate immune defenses, the durable and precise adaptive response is activated. This adaptive immune response involves T cell receptors and B cell receptors, which are presented on the surface of T cells and B cells, respectively [27]. T cell receptors recognize fragments of degraded proteins (peptides) presented by major histocompatibility complex (MHC) molecules. This process heavily relies on antigen-presenting cells (APCs), including DCs, macrophages, and B cells [28,29]. T cell activation commences when they bind to the peptide–MHC complex on APCs, followed by co-stimulation facilitated by DC-bound molecules CD86, OX40L, CD80, and 4-1BBL, leading to the complete activation and functional capacity of the T cell [30,31]. Among T cells, cytotoxic T cells are pivotal in eliminating antigen-expressing cells, such as infected cells, tumor cells, and foreign tissue grafts. Helper T cells aid activating cytotoxic T cells, B cells, and other immune components, while T regulatory cells help distinguish between self and foreign molecules to decrease the risk of autoimmune disease [32]. B cell activation leads to the proliferation of antigen-specific B cells and the differentiation of antibody-producing plasma cells or memory B cells [33]. The secreted antibodies then identify and bind to antigens, assisting in their elimination [34].

The immune response requires precise regulation, and the neddylation pathway appears to be vital in maintaining a well-balanced and finely tuned immune response. Abnormal activation of the neddylation pathway can have substantial impacts on various aspects of both the innate and adaptive immune systems. As detailed below, neddylation affects signal transduction in the innate immune reaction and also influences the activation, differentiation, proliferation, maturation, survival, and functional capabilities of adaptive immune cells. The loss of neddylation function is associated with altered maturation and cytokine release functions of DCs and macrophages, 2 essential types of APCs involved in the adaptive immune response. Furthermore, a number of studies have demonstrated that dysregulated neddylation modification of CRLs is linked to various immune-related diseases [35]. These findings underscore the significance of neddylation in regulating the immune system and suggest that it might serve as a potential target for therapeutic interventions in immune-related disorders.

## Neddylation Regulation of Innate Immune Responses

The innate immune system employs specialized defense cells to combat microorganisms through various mechanisms, including phagocytosis, intracellular killing, recruitment of inflammatory cells, and antigen presentation [18,19]. These defense cells primarily consist of NK cells and phagocytes, including neutrophils, monocytes, tissue DCs, and macrophages. Phagocytes are responsible for recognizing pathogens and destroying them through phagocytosis. This pathogen recognition is often orchestrated through PRRs or the cGAS-STING pathway. Extensive research has revealed that neddylation modification influences not only the function of innate immune cells but also the regulation of PRRs or the cGAS-STING pathway.

### Neddylation regulation of innate immune cells

NK cells are essential effector lymphocytes within the innate immune system. They have a crucial role in identifying and eliminating cancerous or infected host cells to limit the spread of diseases or infections [36]. Inhibition of neddylation by MLN4924 treatment has been shown to enhance the ability of NK cells to release their granules and eliminate multiple myeloma (MM) cells, as well as enhancing the response to Daratumumab/Elotuzumab treatment [37]. The underlying mechanistic study revealed that neddylation suppression leads to increased expression of Rac1 and RhoA GTPases in NK cells [37]. These GTPases are crucial for facilitating the efficient degranulation of cytotoxic lymphocytes. Additionally, it increases the F-actin levels and the perforin polarization in NK cells during target cell interactions [37]. Moreover, neddylation suppression partially counteracts the inhibitory effects of transforming growth factor-β (TGF-β) on effector activity of NK cells by inhibiting the transcriptional activity of SMAD4 through inactivation of the CRL4^AMBRA1^ complex [37]. Besides its direct impact on NK cells, MLN4924, along with a DCN1 inhibitor (NAcM-OPT), has been shown to enhance the cell surface presentation of the NKG2D ligands MICA and MICB on both MM cell lines and patient-derived MM cells. This augmentation renders MM cells more prone to recognition by NK cells, subsequent degranulation, and eventual destruction [38]. Mechanistic investigations further revealed that MLN4924 up-regulates the mRNA level of MICA. This up-regulation is achieved through the inhibition of IRF4 and IKZF3 expression, both of which are key transcriptional repressors of MICA. The reduced IRF4 levels stem from the suppressed activity of the CRL1^βTRCP^–IκBα axis [38]. Another correlation study showed that CUL-5 is negatively associated with the number of NK cells, Tregs, cytotoxic cells, and T helper 17 (Th17) cells in various tumor tissues [39].

Neutrophils, considered “first responders” of the immune response, are crucial in preventing infections by attacking and killing disease-causing microbes [40]. Neddylation inhibition has been shown to affect the number and cytokine production of neutrophils. MLN4924 treatment resulted in elevated levels of neutrophils and monocytes in the bloodstream, but the underlying mechanism remains unknown [41]. Moreover, MLN4924 treatment dose-dependently inhibited the production of proinflammatory cytokines in neutrophils in response to lipopolysaccharide (LPS), while preserving their viability [42]. Further investigations revealed that MLN4924 hindered the degradation and re-synthesis of IκBα in neutrophils, causing an accumulation of p-IκBα in neutrophils, thus dampening the production of tumor necrosis factor-α (TNF-α), IL-6, and IL-1β via inactivation of NFκB [42]. We have explored the role of Sag/Rbx2, a neddylation E3 ligase, in functional regulation of both neutrophils and macrophages by generating *LysM-Cre;Sag^fl/fl^* mice with selective *Sag* depletion in myeloid lineage. We found that mice with *Sag* deletion showed elevated levels of proinflammatory cytokines (such as TNF-α) with enhanced mortality upon LPS exposure. Interestingly, *Sag^−/−^* neutrophils released more proinflammatory cytokines, whereas *Sag^−/−^* macrophages released less. Mechanistically, we found that the *LysM-Cre;Sag^fl/fl^* mice had significant reduction in expression of LPS-responsive genes in bone marrow cells, such as myeloperoxidase, a critical enzyme in neutrophils’ enzyme, and Elane, which is expressed in neutrophils as well. Thus, Sag appears to exert distinct effects on the activation of macrophages and neutrophils [43]. More detailed description of neddylation effect on DCs and macrophages, which serve as professional APCs, will be presented in a later section.

### Neddylation regulation of the signaling pathways related to innate immune responses

#### The PRR family

*TLR signaling*. The TLR family senses a broad array of pathogens both outside the cells (by TLR1, 2, 4, 5, 6, and 10) and intracellularly in endosomes and lysosomes (by TLR 3, 7, 8, and 9), which is critical for the functioning of the innate immune system [44,45]. TLR signaling activation starts from intracytoplasmic TIR domains, and the downstream signaling involves a myeloid differentiation factor 88 (MyD88)-dependent pathway shared among all TLRs. Upon stimulation, MyD88 facilitates the recruitment of IL-1 receptor-associated kinase (IRAK) to TLRs. IRAK is then phosphorylated and interacts with TRAF6, ultimately resulting in the separate activation of JNK and NF-κB separately [46,47].

As a key responder to innate immunity, MyD88 was found to be regulated by neddylation as a new substrate [48]. MyD88 neddylation is enhanced by neddylation E2 UBE2M, but inhibited by deneddylase DENP1. Furthermore, MyD88 neddylation inhibits its ubiquitylation. Interestingly, IL1β inhibits MyD88 neddylation, but enhances MyD88 ubiquitylation. A luciferase-based reporter assay showed that NEDD8 inhibits MyD88-dependent NF-κB activation [48].

During RANKL-mediated osteoclastogenesis and collagen-induced arthritis (CIA), the neddylation pathway was up-regulated, including the up-regulation of UBA3 or NEDD8 and cullin-1. Furthermore, neddylation inhibition by UBA3 knockdown or MLN4924 treatment significantly suppressed RANKL-mediated osteoclastogenesis or CIA responses, respectively. Mechanistically, UBA3-induced neddylation is actively involved in activation of the TRAF6-TAK1-NFATc1 signaling pathway-induced RANKL during osteoporosis and osteoclast differentiation, and TRAF6 neddylation at Lys124 is necessary for NF-κB activation induced by IL-17A in CIA [49,50]. Furthermore, MLN4924 treatment or UBA3 depletion caused the accumulation of phosphorylated IκBα as a result of reduced degradation to block p65 nuclear translocation at the initial phases of herpes simplex virus type 1 (HSV-1) infection, ultimately leading to a decrease in the production of interferon (IFN)-β in the early stages. Although the mechanism by which neddylation regulates the abundance of phosphorylated IκBα was not defined in that study [51], it is likely through inactivation of CRL1^βTrCP^ E3 ligase, which promotes ubiquitylation and degradation of pIκBα [52].

During endotoxin-induced acute lung injury, Cul-5 was found to be necessary for pulmonary inflammation, since the levels of IL-1β, IL-6, TNF-α, CXCL1, and monocyte chemotactic protein-1 (MCP-1) were lower in the lung tissue from Cul-5^+/−^ mice, as compared to wild-type control. Further study showed that Cul-5 regulation of lung inflammation is mediated by alveolar macrophages. Mechanistically, Cul-5 neddylation leads to a direct interaction between the C-terminal domain of Cul-5 and the TRAF-C domain of TRAF6, facilitating TRAF6 polyubiquitination and subsequent activation of NF-κB. As a consequence, proinflammatory cytokines are produced during acute lung injury [53,54]. Finally, in both murine and human DCs, neddylation inhibition suppressed both canonical and noncanonical NF-κB activity by preventing the IκB degradation and subsequent p65 nuclear localization, leading to functional abrogation of DCs [55].

*NLR and RLR signaling*. NLRs, which are essential cytoplasmic receptors, are crucial for the innate immune response through recognizing intracellular PAMPs and DAMPs [56]. The activation of the NLR signaling pathways triggers 4 distinct functional outputs, including inflammasome formation, signaling transduction, transcription activation, and autophagy initiation [56]. Inflammasomes are protein complexes that trigger the self-cleavage of pro-caspase-1, leading to the activation of caspase-1, which, in turn, drives the processing and maturation of proinflammatory cytokines like IL-1β and IL-18. Additionally, inflammasome activation is associated with an inflammatory form of cell death, known as pyroptosis [57,58]. NEDD8 plays a crucial role in facilitating the efficient self-cleavage of pro-caspase-1 to generate activated caspase-1[59]. Specifically, NEDD8 silencing or MLN4924 treatment has been shown to decrease the caspase-1 processing and subsequent IL-1β maturation, while enhancing the auto-catalytic activity of pro-caspase-1 in 293 cells upon NEDD8 overexpression. Mechanistically, NEDD8 promotes the neddylation of the CARD domain of pro-caspase-1, facilitating its auto-cleavage and activation, resulting in mature IL-1β release during inflammasome activation [59].

The cytosolic RLRs play a crucial role in intracellular immune surveillance against viral infections by sensing viral RNA ligands or processed self-RNA, which drives the production of type 1 IFN and the expression of antiviral gene [60]. Three RLR members have been identified: RIG-I, melanoma differentiation-associated protein 5 (MDA5), and laboratory of genetics and physiology 2. Once activated, RIG-I and MDA5 interact with the mitochondrial antiviral-signaling protein, relaying the signal to TANK-binding kinase 1 (TBK1) and IκB kinase-ε, leading to the activation of interferon regulatory factor 3 (IRF3) and IRF7, and ultimately the expression of type I interferons and the other genes.

Our collaborative study showed recently that macrophages with Ube2m depletion had decreased IFN-I expression upon RNA virus infection in a RIG-I-dependent manner, resulting in an aggravated viral infection [61]. A mechanistic study revealed that UBE2M inhibits RIG-I degradation by preventing RIG-I binding to STUB1 E3 ligase, resulting in activation of antiviral IFN-I signaling, which then activates STAT1 to transactivate the expression of Trim21, an E3 ligase that promotes UBE2M ubiquitylation and degradation, resulting in inhibition of antiviral immunity. Thus, blockage of this negative feedback loop of the IFN-I signal to retain high levels of UBE2M would increase innate immunity against RNA viruses [61].

During the infection caused by the spring viremia of carp virus (SVCV), IRF3 and IRF7 were reported to undergo neddylation, suggesting their involvement in the viral response [62]. In the zebrafish model, neddylation inhibition by MLN4924 suppressed antiviral response. Nedd8 depletion sensitized zebrafish to SVCV infection. Mechanistically, blockage of neddylation markedly suppressed the expression of crucial antiviral genes following either poly (I:C) stimulation or SVCV infection with an increase in SVCV replication. Increased neddylation of Irf3 and Irf7 was detected, but the underlying mechanism by which neddylation E2 or E3 was not defined. Nevertheless, the study showed that neddylation indeed facilitates the antiviral response [62].

During Sendai virus (SeV) infection, neddylation also plays a role in IRF-3 degradation. Inhibition of neddylation E1 stabilizes IRF-3. The mechanistic studies revealed that IRF3 degradation results from the C-terminal phosphorylation by polyubiquitinated TBK1, which is induced by neddylated cullin-1 [63]. In another study involving the infection of SeV or influenza A H1N1 virus, the deficiency of UBA3 or NEDD8 in myeloid cells impaired IFN-α production and render mice more susceptible to RNA virus infection. The mechanistic study suggested that neddylation directly affects IRF7, enhancing its transcriptional activity by facilitating its nuclear translocation and inhibiting its dimerization with IRF5, an IFN-α repressor when interacting with IRF7. This is particularly important as IRF5 can act as a repressor of IFN-α when it interacts with IRF7 [64]. Furthermore, treatment with MLN4924 had a significant dose-dependent effect, impairing the production of IFN-β induced by LPS, poly(I:C), or SeV at both protein and mRNA levels. Mechanistically, MLN4924 inhibits the transcriptional activation of IRF3 and prevents IRF3 from binding to the IFN-β promoter, thereby reducing IFN-β production. However, NEDD8 knockdown and SENP8 overexpression had no impact on IFN-β expression, suggesting that MLN4924-induced reduction of IFN-β production might be neddylation-independent [65]. The UBA3 knockdown experiment will validate whether neddylation modification is indeed involved in this process, or merely one of off-target effects of MLN4924 [11].

#### The cGAS-STING pathway

The cGAS-STING pathway drives innate immune activation by detecting and interacting with cytosolic DNA [66,67]. Cytosolic DNA activates cGAS, leading to the generation of 2′3′-cGAMP, which, in turn, binds to the ER adaptor protein STING. This interaction results in TBK1-dependent phosphorylation of IRF3, which then forms dimer and entries into the nucleus to transactivate the expression of type I IFN and other immunomodulatory molecules [25]. Our collaborative study has highlighted the importance of neddylation modification in regulating cGAS-STING signaling activation during HSV infection. Specifically, MLN4924 treatment or UBA3 knockdown reduced the mRNA levels of Ifnb, Ifna4, and Cxcl10, as well as the production of IFNβ, CXCL10, and IFIT2. Mechanistically, neddylation E2, UBE2M, but not UBE2F couples with RNF111 E3 polyneddylated cGAS at the multiple sites, promoting cGAS dimerization and enhancing its DNA-binding activity. This ultimately led to proper activation of the cGAS-STING pathway and type 1 IFN release for effective defense against HSV infection. Consistently, mice deficient in either Ube2m or Rnf111 displayed severe defects in innate immune response and were susceptible to HSV-1 infection [68].

**Fig. 2.**
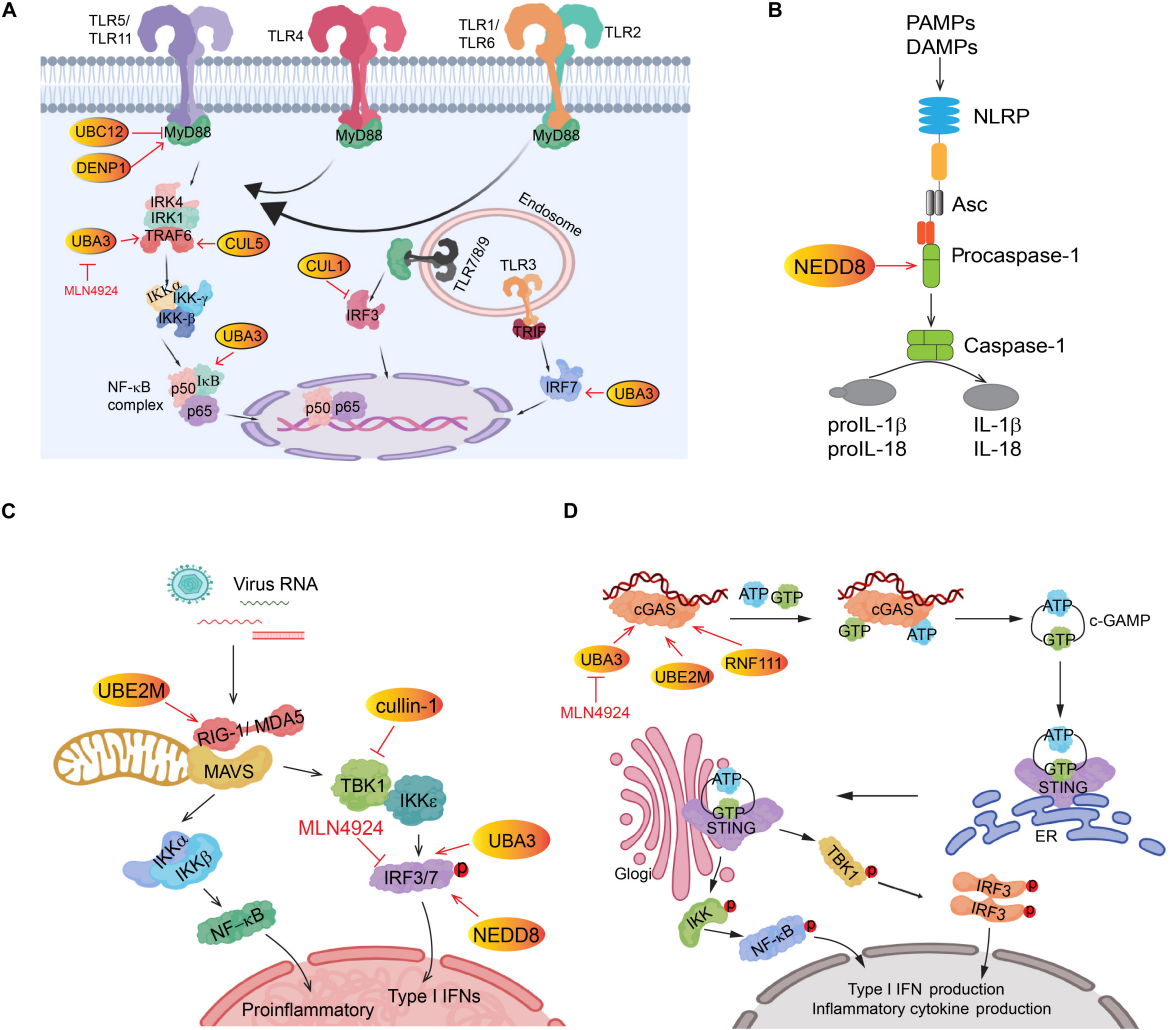
Neddylation regulates the innate immune responses. Neddylation serves as a crucial regulator of signaling pathways associated with the innate immune responses. (A) Key components of neddylation, such as UBE2M, DENP1, UBA3, CUL1, and CUL5, operate at various points along the TLR signaling pathway, ultimately converging at NFκB activation. (B) Neddylation also facilitates inflammasome activation by promoting the neddylation of pro-caspase-1. (C) NEDD8, UBA3, UBE2M, and Cullin-1 participate in RLR signaling activation. (D) UBA3, UBE2M, and RNF111 contribute to the activation of the cGAS-STING pathway. Images are created with MedPeer (www.medpeer.cn).

In summary, neddylation modulates the innate immune system at multiple points, affecting both innate immune cells and regulating multiple signal pathways (Fig. 2). However, it is worth noting that some observations made in this section were based on the use of MLN4924. Given that MLN4924 has demonstrated several neddylation-independent off-target activities [11], more genetically modified mouse models, via targeting neddylation E1, E2s, or E3s, respectively, in innate immune cells, should be utilized to provide more definitive answers as to whether and how neddylation affects the innate immune system and its responses, leading to translational implications for potential therapeutic interventions.

## Neddylation Regulation of Maturation, Polarization, Function, and Survival of APCs

**Fig. 3.**
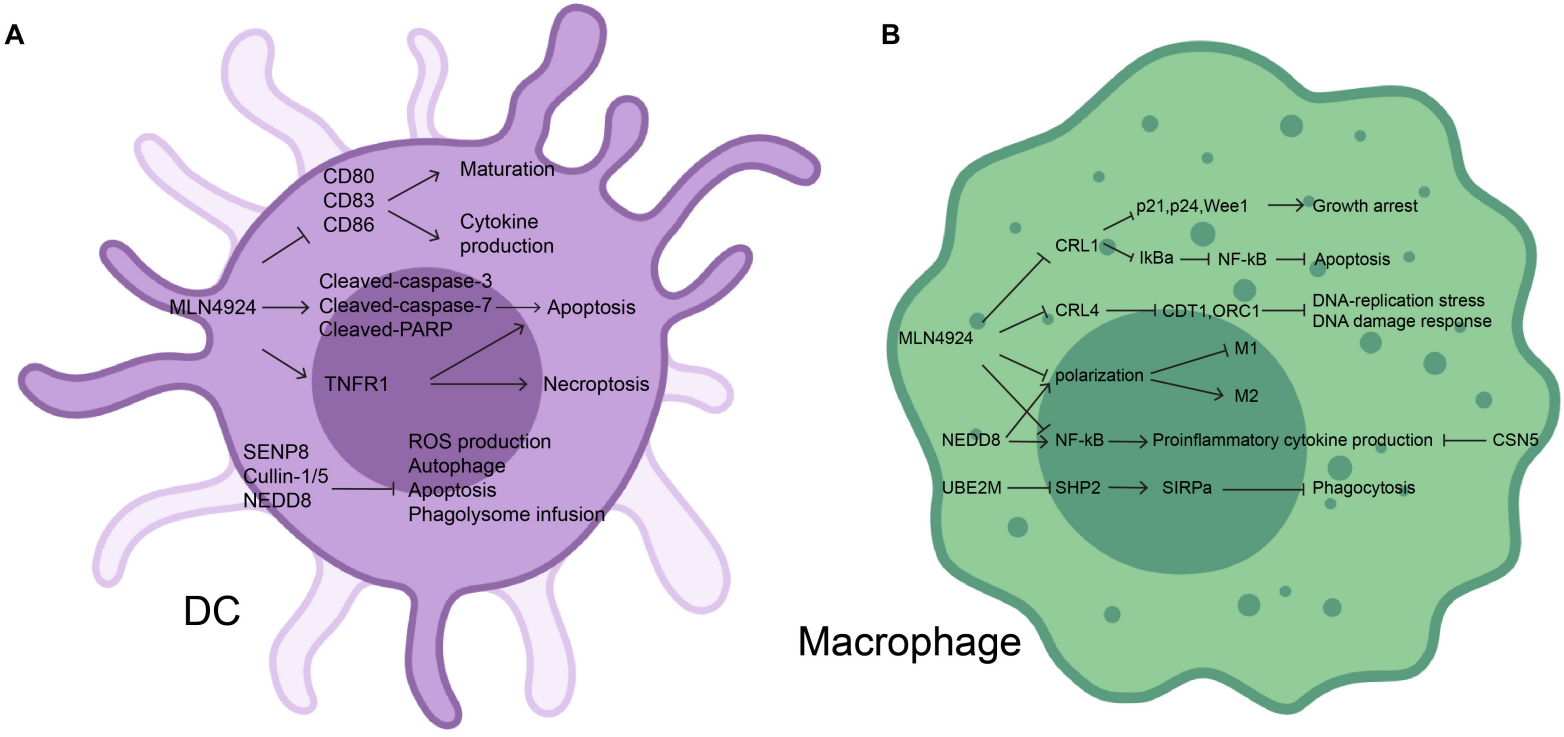
Neddylation regulates antigen-presenting cell (APC) functions. Neddylation plays a critical role in governing the maturation, polarization, function, and viability of APCs. (A) Effect on dendritic cells (DCs): Neddylation orchestrates diverse mechanisms that regulate the maturation, cytokine production, phagocytosis, and survival of DCs. (B) Effect on macrophages: Neddylation exerts multifaceted effects on macrophages, regulating their polarization, cytokine production, phagocytosis, and survival. Images are created with MedPeer (www.medpeer.cn).

Antigen processing and presentation form the basis for adaptive immunity, encompassing multistep processes. An APC phagocytizes a harmful organism, such as a virus or bacteria, to initiate the process. The engulfed organism is then digested within the acidic lysosomes of the APC, and proteins from the organism are broken down into peptides inside the lysosome. The peptide antigens form a complex with MHC molecules in a vesicle, and the MHC–peptide complex is subsequently displayed on the surface of the APC. Finally, the APC presents the peptide antigen to naive T cells [69,70]. Primary professional APCs consist of DCs, macrophages, and B cells [71,72]. These cells have a vital role in capturing, processing, and presenting antigens to initiate and regulate the adaptive immune response. It seems that neddylation modification is essential for the function of APCs (Fig. 3).

Apart from functioning as the most efficient APCs to activate cytotoxic and helper T cells for controlling infections and cancer, DCs are also essential in preserving the immune tolerance to self-antigens [73]. Mature DCs present foreign antigens to naive T cells, inducing an efficient T cell response required for controlling the infection [74,75]. The maturation of DCs is characterized by a decrease in endocytic activity and up-regulation of class I and II molecules and co-stimulatory molecules like CD80 and CD86 on their surface. The surface expression of these costimulatory molecules in DCs is regulated in a neddylation-dependent manner [76]. In the context of inflammatory bowel disease (IBD), the neddylation inhibitor MLN4924 has been shown to have therapeutic efficacy in a murine IBD model. MLN4924 treatment leads to a dose-dependent decrease in the expression levels of activation markers CD80, CD83, and CD86 in DCs, thus dampening their maturation and release of cytokines, such as IL-6, TNF-α, and IL-12, which suppress the stimulation and proliferation of allogeneic T cells by DCs. Mechanistically, MLN4924 inhibits CUL-1 neddylation to inactivate CRL1 E3, leading to Deptor accumulation and subsequently inactivation of mTORC1/2, or IκB accumulation to inactivate the classical NF-κB pathway [76–78]. Moreover, the neddylation pathway plays an indispensable role in the survival of DCs. Inhibition of neddylation by MLN4924 induces caspase-dependent apoptosis, likely through the CUL-1–Deptor–mTOR (mammalian target of rapamycin) axis and enhances TNF-induced apoptosis and necroptosis of immature DCs (iDCs) [76,77]. In a separate study, it was observed that when *Mycobacterium tuberculosis* (M. tb) infected the body, there was a decline in NEDD8 expression within DCs, coupled with an elevation in SENP8 levels. The M. tb antigens Rv2463 and Rv3416 stimulate the binding between NEDD8 and CUL-1. Interestingly, knockdown of NEDD8, CUL-1, or CUL-5 increased the ROS levels induced by Rv2463, and promoted phagolysosome fusion in infected DCs, eventually inducing cell autophagy and apoptosis [79,80]. These results align with an earlier study, showing that SENP8 knockdown triggers pro-inflammatory T cell responses to induce autophagy in DCs [80].

As another crucial APC, macrophage is involved in the detection, phagocytosis, and destruction of microorganisms. Macrophages are a heterogeneous and versatile group of cells [81]. Currently, 2 major macrophage subpopulations have been identified, known as pro-inflammatory (M1) and anti-inflammatory (M2) macrophages [82]. M1 macrophages undergo polarization in response to LPS, either independently or in combination with Th1 cytokines, and they produce pro-inflammatory cytokines to combat and destroy pathogens. On the other hand, M2 macrophages receive polarization signals from Th2 cytokines, and they generate anti-inflammatory cytokines to aid in the repair of inflammation-associated injuries [83–85]. However, the process of macrophage polarization is highly plastic [86].

It was reported that neddylation regulates macrophage polarization, which is, however, influenced by the cell type and microenvironment. MLN4924 treatment promotes the polarization of M2 macrophages while decreasing M1 differentiation in bone marrow-derived macrophages. This is characterized by an increase in M2 markers, such as arginase-1, IL-13, CD11b+, and CD206, and a decrease in M1 markers, including TNF-α, IL-6, F4/80, and IL-12 [87]. In another study, neddylation inhibition by MLN4924 was reported to reduce LPS-induced inflammation, and favors an anti-inflammatory macrophage phenotype, leading to a delayed progression of early atherosclerotic lesions in mice [41]. On the other hand, neddylation enhancement by depletion of deneddylase Csn5 potentiates the expression of pro-inflammatory cytokines in macrophages, exacerbating atherosclerotic lesions with the mechanism involving the modulation of NF-κB, hypoxia inducible factor 1 subunit alpha (HIF-1α), and MAPK signaling pathways [41].

In a model of metastatic lung cancer, CRISPR/Cas9-based NEDD8 depletion remarkably reduced the population of both M1 and M2 types of macrophages [88]. Additionally, the neddylation pathway also affects macrophage releasing of inflammatory cytokines. Neddylation blockage, either by MLN4924 or by knockdown of NEDD8 or neddylation E2 UBE2M/UBC12, affects NF-κB translocation and hinders the production of inflammatory cytokines such as IL-6, TNF-α, and IL-1β in response to LPS stimulation [41,89,90]. Likewise, MLN4924 treatment notably reduced the levels of several cytokines, including IL-6, IL-18, TNF-α, IFN-γ, IL-1α, and IL-1β in RAW264.2 macrophages [87]. These findings underscore the crucial role of neddylation in modulating the inflammatory response of macrophages.

Our collaborative study recently showed that neddylation promotes macrophage-mediated phagocytosis via modulating the neddylation status of SHP2 (SH2 containing protein tyrosine phosphatase-2). Specifically, UBE2M, but not UBE2F, promotes SHP2 neddylation, whereas deneddylase SENP8 deneddylates SHP2. Neddylation deactivates SHP2, facilitating the ability of macrophages to engulf opsonized tumor cells and enhances the effectiveness of in vivo immunotherapy when combined, whereas SENP8 maintains SHP2 in a deneddylation status to activate and recruit it toward SIRPα, which, in turn, suppresses macrophage phagocytosis [91]. This illustrates the fine-tuned and balanced role of neddylation in regulating macrophage functions.

Finally, the neddylation pathway is essential for the proliferation and survival of macrophages and its inhibition triggers growth suppression and apoptosis induction through 3 mechanisms. First, neddylation inhibition by MLN4924 blocks cullin neddylation, suppressing CRL1 activity and causing the accumulation of cell cycle inhibitors like p21, p27, and Wee1, resulting in cell cycle arrest at both the G1 and G2/M phases. Second, neddylation inhibition inactivates CRL1, also causing accumulation of IκBα to retain NF-κB in the cytoplasm, leading to its inactivation and apoptosis induction. Third, it inactivates CRL4 to cause the accumulation of DNA replication licensing proteins, including CDT1 and ORC1, thereby activating DNA re-replication stress and DNA damage responses [90,92].

In summary, neddylation plays a crucial and positive role in the maturation, polarization, pro-inflammatory function, and survival of APCs. These findings suggest that neddylation is necessary for T cell activation, which relies on the normal function of APCs. Exploring how neddylation influences APCs and its repercussions on T cell reactions has the potential to yield valuable insights for fine-tuning immune responses and devising therapeutic approaches to address a range of immune-related conditions.

## Neddylation Regulation of Adaptive Immune Response

The adaptive or acquired immune system, executed by distinct categories of lymphocytes known as B cells and T cells, comes to play when the innate immune response falls short of managing an infection [27]. Activated T cells coordinate the cellular arm of the immune response, whereas activated B cells, along with antibodies they generate, trigger the humoral immune response. Through the collaborative efforts of T cells and B cells, invading pathogens and their associated toxic molecules face eradication [93]. A wealth of research has underscored the significance of neddylation in regulating adaptive immune responses.

### Neddylation regulation of T cell activation, differentiation, function, and survival

T cells hold a pivotal position in the realm of adaptive immunity. They originate in the bone marrow before proceeding to the thymus for maturation and differentiation into CD4+ or CD8+ T cells [32]. Upon encountering foreign antigen peptide-MHC complexes presented on APCs along with CD4/CD8 receptors and costimulatory signals like CD28 in lymphoid organs, naïve CD4+ or CD8+ T cells become activated [94]. The TCR/CD28 co-stimulation triggers the activation of the Lck-ZAP70-LAT/SLP-76 signaling pathway, leading to the activation of phospholipase C γ 1 and downstream protein kinase Cθ, the RAS/MEK/ERK, and calcium pathways. This ensures subsequent T cell proliferation, cytokine production, differentiation into various subsets, and survival [95].

**Fig. 4.**
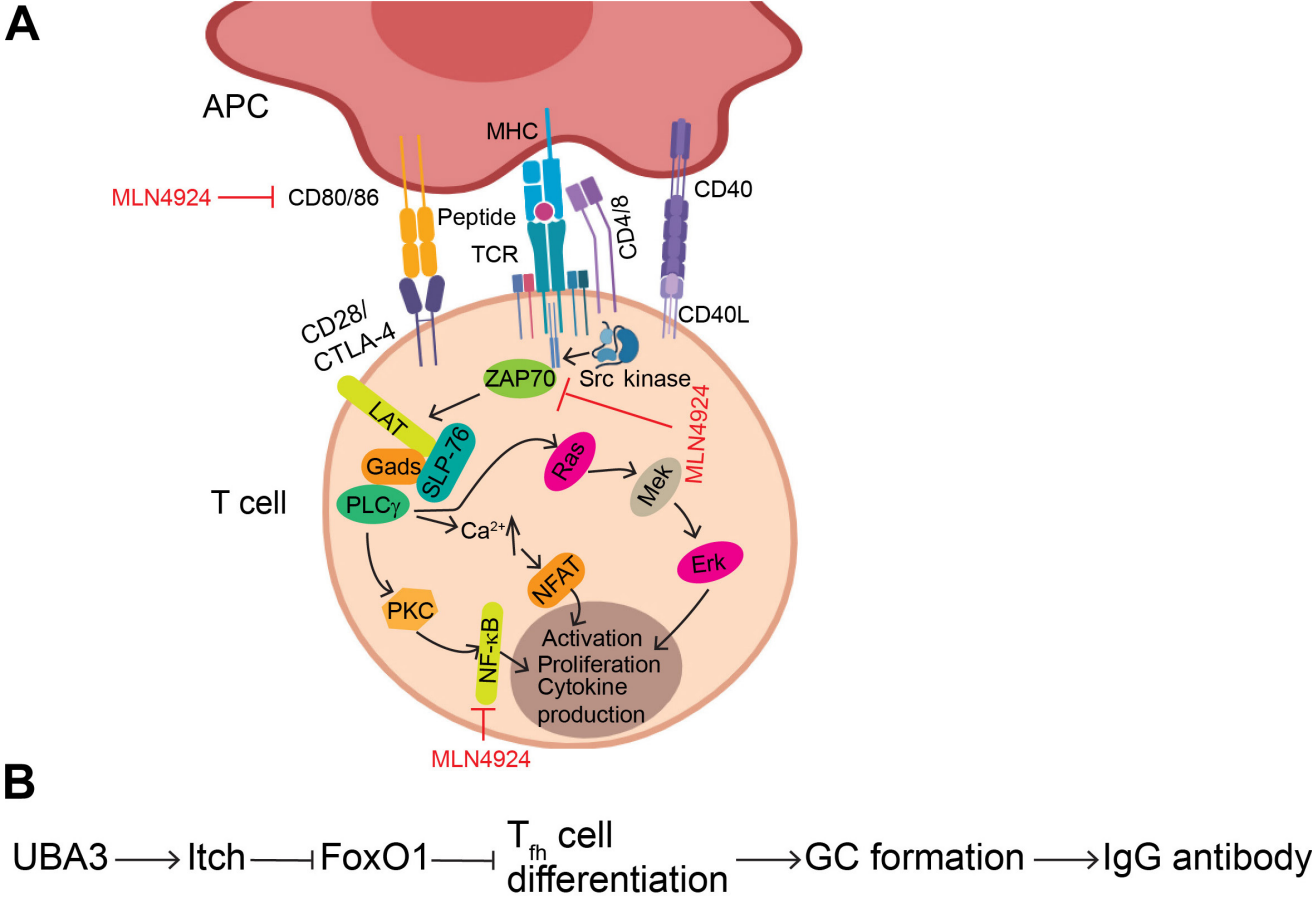
Neddylation regulates adaptive immune responses: (A) Neddylation governs T cell activation by orchestrating various mechanisms: (a) MLN4924 down-regulates the expression of activation markers CD80 and CD86, and consequently attenuates the maturation and cytokine production of dendritic cells; (b) MLN4924 halts the nuclear translocation of NF-κB; and (c) MLN4924 impedes the formation of a ZAP70–Shc–Grb2 signaling complex. (B) Neddylation regulation of B cell functions: UBA3 regulates germinal center (GC) formation and antibody production through its impact on the differentiation of Tfh cells. Images are created with MedPeer (www.medpeer.cn).

Emerging evidence indicates that neddylation regulates the activation, proliferation, differentiation, cytokine production, and survival of T cells (Fig. 4). Our collaborative study showed that neddylation disruption by MLN4924 or T cell selective Sag knockout (via Lck-Cre) up-regulates the suppressor of cytokine signaling (SOCS) proteins, specifically SOCS1 and SOCS3 with minimal effect on the NF-κB translocation in T cells. Consequently, this potently reduces T cell activation, proliferation, and the release of cytokines both in vitro and in vivo, leading to a marked reduction in graft-versus-host disease in recipient mice [96].

**Fig. 5.**
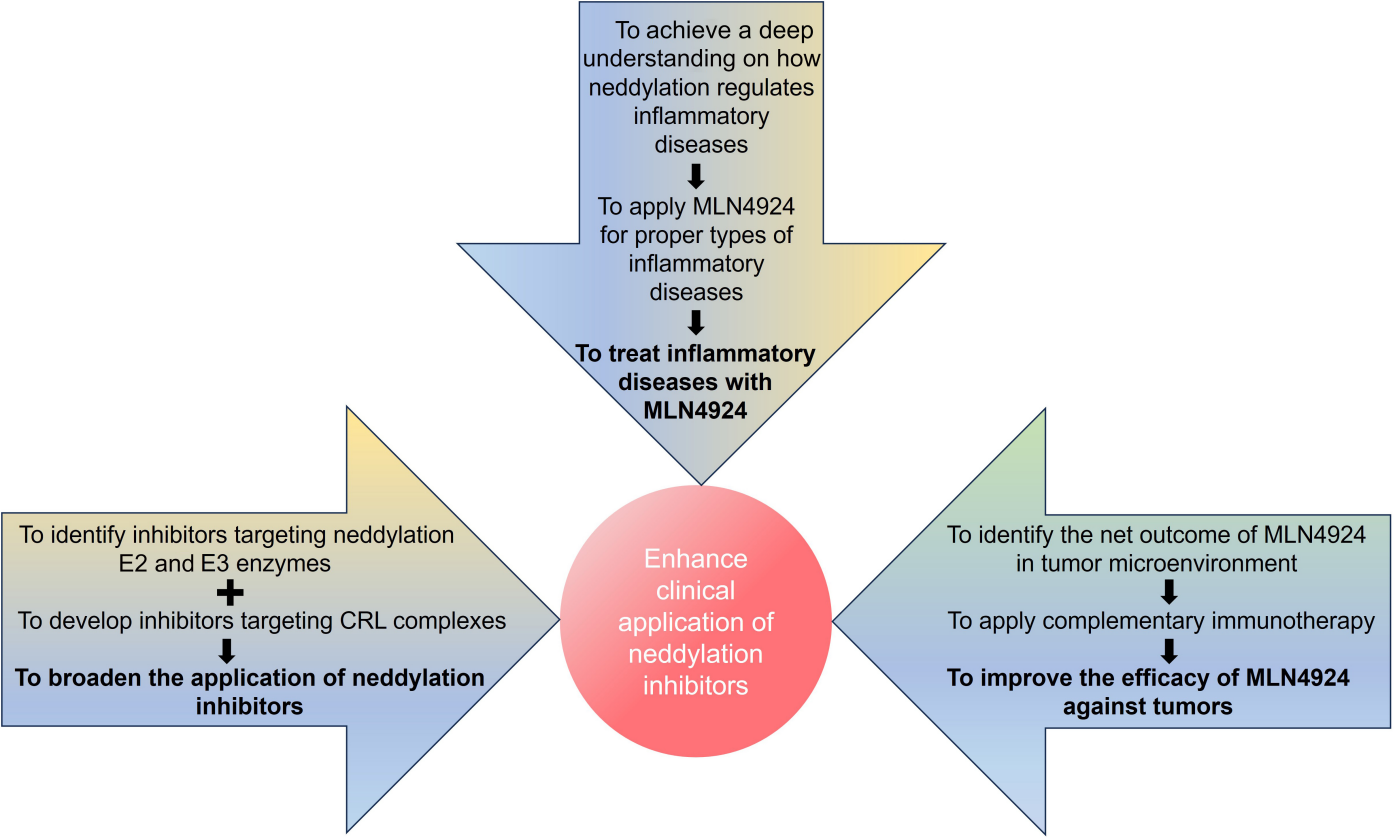
Proposed future perspectives. See the main text for details.

Similarly, MLN4924 treatment or UBE2M/UBC12 knockdown in CD4+ T cells inhibits cell proliferation and reduces the production of cytokines like IL-2, IL-4, and IFN-γ, and ERK activation. Biologically, mice adoptively transferred with Ube2m knockdown CD4^+^ T cells displayed significantly improved responses to allergies. Mechanistically, neddylation of Shc might enhance the formation of a ZAP70–Shc–Grb2 signaling complex, affecting downstream ERK activation and influencing T cell proliferation [97]. On the other hand, another study showed that MLN4924 treatment increased IFN-γ production in human chronic lymphocytic leukemia (CLL) T cells [98], suggesting that MLN4924 regulation of IFN-γ production is cell-type dependent. Furthermore, after TCR stimulation, both total and neddylated Cul-4b levels are up-regulated, and CUL-4b plays a vital role in supporting the proper proliferation and survival of activated CD4+ T cells, and CD4+ T cells lacking Cul-4b are unable to repair DNA damage [99]. These findings demonstrate the intricate and context-dependent role of neddylation in regulating responses and functions of T cells.

In a mouse model of malaria infection induced by *Plasmodium yoelii* 17XNL, the protein levels of Uba3 are induced as a cellular protective response. Selective Uba3 knockout in T cells remarkably increases the susceptibility of mice to *P. yoelii* 17XNL infection. Using both conditional Uba3 and Nedd8 knockout models, neddylation was shown to be required for activation, proliferation, survival (via anti-apoptotic protein Bcl-2), and IFN-γ production, and promotes the formation of defensive T helper 1 (Th1) cell response within CD4+ T cells. Neddylation also enhances the differentiation of follicular helper T (T_fh_) cell, possibly by activating the ubiquitin ligase Itch to promote proteasomal degradation of FoxO1 [100,101]. Furthermore, neddylation has immunomodulatory effects. For example, MLN4924 treatment induced the accumulation of pIκBα, preventing nuclear translocation of RelA/p65, resulting in inhibition of the activation, proliferation, and release of IL-2 by T cells, eventually leading to reduced Treg genesis and a shift to the TH1 phenotype with increased production of IFN-γ production in human CLL T cells [98].

Finally, neddylation also regulates the function and fitness of Treg cells. We recently systematically investigated the role of neddylation on Treg cells by generating 4 lines of conditional knockout mice models, targeting 2 E2s, Ube2m and Ube2f, and 2 E3s, Rbx1 and Sag, individually, driven by Foxp3-Cre [102]. It is well established that UBE2M E2 collaborates with RBX1 E3 to perform neddylation on cullins 1 to 4, whereas UBE2F E2 partners with SAG/RBX2 E3 to facilitate neddylation on cullin 5, resulting in activation of CRL1 to CRL4 or CRL5, respectively [103]. We found that while Ube2f or Sag knockout in Treg cells showed no phenotype, Rbx1 Treg knockout triggers severe autoimmune phenotypes, leading to an early-onset fatal inflammatory disorder, and a similar inflammatory phenotype, but, to a lesser extent, was also seen in Ube2m Treg knockout mice [102]. Mechanistic studies revealed activation of many inflammatory signal pathways and accumulation of pro-apoptotic Bim, a CRL substrate [104] in Rbx1-null Treg cells. However, simultaneous Treg knockout of Rbx1 and Bim showed minor rescue of inflammatory phenotype, indicating involvement of many other signal molecules or CRL substrates [102]. Most recently, we showed functional redundancy of 2 neddylation E2s and E3s, since simultaneous Treg double knockout of Ube2m and Ube2f produces much severer phenotypes than Ube2m Treg single knockout. Similarly, Treg double knockout of Rbx1 and Sag also produces severer phenotypes than Rbx1 Treg single knockout, although to a much lesser extent. Thus, either Ube2f or Sag actually plays a role in the functional maintenance of Treg cells, but their effects were fully compensated by Ube2m or Rbx1, respectively [105]. The fact that a stronger phenotype severity upon Treg KO of downstream Rbx1 was observed than that of upstream Ube2m [102], and upon Treg KO of double E3s than double E2s [105], strongly suggests an additional mechanism, distinct from neddylation, played by these 2 E3 enzymes, also known to act as the RING components of CRLs in the functional regulation of Treg cells [105,106].

Collectively, these results suggest that neddylation plays a critical role in regulating multiple aspects of T cell function, including activation, proliferation, differentiation, survival, cytokine production, and effector function. It appears that neddylation modification exerts a dual role in T cell-mediated immune reactions, but the specific outcomes may vary in a manner dependent of cellular context. Consequently, when considering the use of MLN4924 or other neddylation inhibitors in therapeutic treatments, such as cancer, it is essential to carefully monitor the immune responses of patients to ensure optimal outcomes and minimize any potential adverse effects.

### Neddylation regulation of B cell functions

B cells are another major cell type for adaptive immunity, although they also serve as professional APCs [29]. B cells originate from the bone marrow and develop in an ordered maturation and selection process. High-affinity antibody-producing B cells are generated and chosen within the germinal center (GC), aided by TH cells [107]. Moreover, the majority of human B cell lymphomas, such as diffuse large B cell lymphoma (DLBCL), trace their origins back to the GC [108].

It was reported that neddylation boosts the formation of GCs and the production of parasite-specific antibodies by enhancing the differentiation of T_fh_ cell, crucial facilitators for the GC reaction and T-dependent humoral immunity [100,109]. In comparison to Uba3^fl/fl^ littermates, Uba3ΔT mice infected with *P. yoelii* 17XNL displayed reduced immunoglobulin G (IgG) levels, a significant decrease in the number of PNA+ GCs, along with severe disruption of their structure within B cell follicles. Additionally, there was a decrease in CXCR5+PD-1+T_fh_ cells and reduced expression of IL-21 and Bcl-6, which are characteristic cytokines and a key transcription factor, respectively, of the T_fh_ lineage. This process may be mediated by the up-regulated activity of ubiquitin ligase Itch and proteasomal degradation of FoxO1 [100].

In addition, a few studies have also shown how MLN4924 inhibits the growth and survival of neoplastic B cells. For instance, MLN4924 induces apoptosis in CLL B cells and reduces drug resistance by causing accumulation of (a) phospho-IκBα through CRL inactivation, blocking the nuclear translocation of p65 and p52 and, thus, inhibiting both the canonical and non-canonical NF-κB pathways; and (b) NOXA and BIM, eventually leading to apoptotic cell death [110]. Moreover, MLN4924 promotes apoptosis of proliferating CLL B cells under IL-21 and CD40L stimulation and sensitizes CLL cells to alkylating agents bendamustine and chlorambucil [111]. Mechanistically, MLN4924 up-regulated CDT1 to accumulate CHK1 and CHK2, thus inducing DNA damage, checkpoint activation and cell cycle arrest, and the final apoptosis of CLL B cells [111]. Similarly, MLN4924 sensitizes DLBCL and CD40-stimulated primary CLL cells to extrinsic apoptosis through promoting TRAIL-mediated caspase-8 activation [112]. Collectively, these studies underscore the role of neddylation in governing the functions of B cells (Fig. 4).

## Neddylation Regulation of Inflammatory Diseases

Inflammatory diseases encompass a wide range of disorders characterized by inflammation. These include autoimmune diseases (AIDs), hepatitis, asthma, IBD, and transplant rejection, among others [113–115]. It has been shown that neddylation modification plays dual roles in inflammation due to its impact on immune responses. Deep mechanistic understanding of neddylation regulation of immune processes would provide precise insights into the pathogenesis and potential therapeutic approaches for potential treatment of these inflammatory conditions.

### Neddylation regulation of autoimmune diseases

AIDs encompass more than 80 different conditions with a common pathogenesis, where the immune system produces autoantibodies or triggers autoreactive immune cells to target and harm the body’s own tissues, leading to pronounced inflammatory reactions and damage to various organs, including the skin, joints, the renal system, and the nervous system, among others [116]. While there is no study in the literature reporting a direct regulation of NEDD8 or neddylation enzymes on the development of AIDs, CRLs, whose activity was activated by neddylation, have been found to play an important role in several AIDs, such as psoriasis, type I diabetes, systemic lupus erythematosus (SLE), multiple sclerosis, and rheumatoid arthritis (RA) [35]. In addition, the neddylation inhibitor MLN4924 was also shown to regulate some AIDs by inactivating CRLs. Thus, it is evident that some AIDs are indeed subject to neddylation regulation.

In a mouse model of psoriasis, MLN4924 treatment inactivates CRL4 and worsened the severity of the disease [117]. Similarly, depletion of CRL4^DCAF2^ accelerated psoriasis in mice. Mechanistically, MLN4924 treatment or DCAF2 deficiency led to the accumulation of NIK protein, which activated the non-canonical NF-kB signaling pathway, resulting in increased IL-23 release and promoting the progression of psoriasis [117]. In individuals with multiple sclerosis, an autoimmune disease affecting the central nervous system, there was a significant increase in the expression of NAE1 in CD4+ T cells. Notably, in a mouse model of experimental autoimmune encephalomyelitis, MLN4924 effectively ameliorated disease severity [118].

Compared to healthy individuals, transcription factors Aiolos and Ikaros, which are degraded by CRL4^CRBN^, were notably higher in SLE patients. Consequently, a new modulator of CRBN, Iberdomide (CC-220), facilitated the interaction between Aiolos and Ikaros and CRBN E3 ligase. This, in turn, resulted in reduced cell proliferation, diminished differentiation into plasma blasts, and a decrease in the secretion of IgG by B cells, eventually an improvement in SLE symptoms [119,120]. Furthermore, in a mouse model of SLE induced by tetramethylpentadecane (TMPD), CRL1^fbxw7^ was found to induce apoptosis of peritoneal macrophages and neutrophils, leading to aggravated symptom in wild-type mice, as compared to myeloid cell-specific Fbxw7-depleted mice [121], further supporting the notion that neddylation-CRLs regulate the progression of SLE.

In the case of RA, CUL-4B expression was dramatically up-regulated in a rat RA model. Elevated Cul-4B promoted the canonical Wnt signaling activation and the pro-inflammatory cytokine production, such as IL-1β and IL-8, thus worsening the pathogenesis of RA [122]. On the other hand, CRL4^CRBN^ was found to promote the ubiquitination and degradation of c-Jun, leading to the suppression of pro-inflammatory cytokines like COX-2, iNOS, IL-1β, and IL-6, ultimately attenuating the symptoms of RA [123]. These contrasting findings highlight the complex and diverse roles that neddylation plays in different or even the same autoimmune diseases.

### Neddylation regulation of other inflammatory diseases

Emerging evidence shows that neddylation also plays a role in various inflammatory diseases, including asthma, IBD, and transplant rejection [35,53,96,124,125]. Two studies showed that, in animal models of asthma and other AIDs, an IL-2–NAEi protein drug conjugate that inhibits cullin5 neddylation showed significantly better efficacy than just using low-dose IL-2 in suppressing the progression of autoimmunity and asthma [124,126]. Another study showed that neddylation inhibition protected mice from mucosal inflammation via inhibiting DC maturation to reduce the production of cytokine and costimulatory molecules, thus suppressing capacity in allogeneic T cell stimulation via a mechanism involving accumulated Deptor to inactivate mTOR, as a result of CRL1 inactivation [76]. Consistently, increased neddylation due to the loss of deneddylase-1 (SENP8) has been linked to the onset of mucosal inflammatory diseases in both mice and individuals with IBD [125]. Furthermore, an elevated expression level of CRL1^fbxw7^ has been observed in both human and murine cases of IBD. Consequently, during dextran sodium sulfate (DSS) or 2,6,4-trinitrobenzene sulfonic acid (TNBS)-induced colitis, the depletion of Fbxw7 in myeloid cells has been shown to mitigate colitis. Mechanistically, Fbxw7 promotes the ubiquitination and degradation of EZH2, leading to the suppression of H3K27 methylation mediated by EZH2 at the gene promoters of Ccl2 and Ccl7. This results in increased production of CCL2 and CCL7 by resident CX3CR1hi macrophages, ultimately leading to heightened recruitment of proinflammatory macrophages to the local inflammatory colon [127].

Moreover, mice adoptively transferred with Sag knockout T cells exhibited significantly decreased graft-versus-host disease, as Sag knockout led to reduced T cell proliferation and decreased secretion of effector cytokines [96].

Notably, inflammation is intricately linked to obesity and the associated metabolic disorders [128]. Our collaborative study showed recently that Ube2m plays a critical role in obesity-related inflammation, triggered by macrophages. When mice had Ube2m depleted specifically in macrophages using Lyz-Cre, the development of obesity, insulin resistance, and hepatic steatosis caused by a high-fat diet was significantly mitigated. This improvement was attributed to the reduced proinflammatory activity of Ube2m-null macrophages, which resulted in a decrease in the production of IL-1β. A mechanistic study revealed that UBE2M depletion inhibits the neddylation of E3 ubiquitin ligase TRIM21, and reduced TRIM21-mediated ubiquitylation and degradation of von Hippel-Lindau (VHL). Accumulated VHL then promotes ubiquitylation and degradation of HIF-1α to reduce IL-1β production. Thus, targeting macrophage UBE2M may have therapeutic value for the treatment of inflammation-induced obesity and associated metabolic diseases [129].

Finally, in an LPS-induced acute kidney injury (AKI) model, MLN4924 attenuates renal inflammation by inactivating CRL1 and subsequent NFκB inactivation, leading to reduced production of pro-inflammatory cytokines (TNF-α, IL-6, and IL-1β) upon LPS stimulation in renal tubular cells [130]. Similarly, in the context of LPS-induced acute lung injury (ALI), Cul-5 depletion significantly reduced lung injury. Mechanistic studies revealed that Cul-5 neddylation upon LPS exposure induced an interaction between Cul-5 and TRAF6, leading to the ubiquitination of TRAF6, which, in turn, activated NF-κB to generate proinflammatory cytokines [53]. On the other hand, depletion of Ube2m or Rbx1 in Treg cells impaired the immunosuppressive function, triggering a severe inflammatory response [102]. Thus, the neddylation may have opposite roles depending of the cellular context. The neddylation regulation of inflammatory diseases is summarized in Table.

**Table.** Neddylation affects the development of inflammatory diseases

Diseases	Neddylation-related mechanism	The role of neddylation in disease progression	Neddylation-related inhibitor
Psoriasis	MLN4924⊣CRL4^DCAF2^⊣NIK→NF-κB→IL-23	Inhibition	MLN4924 [117]
Multiple sclerosis	NAE1 is up-regulated in CD4+ T cells	Acceleration	MLN4924 [118]
SLE patient	CRL4^CRBN^⊣Aiolos and Ikaros	Inhibition	CC-220 [119,120]
TMPD-induced SLE	CRL1^fbxw7^ is necessary for the progression	Acceleration	[121]
RA	1. CUL-4B→Wnt→IL-1β and IL-8	Acceleration or inhibition	[122,123]
	2. CUL4^CRBN^⊣c-Jun→ COX-2, iNOS, IL-1β, IL-6		
Asthma	Cullin 5	Acceleration	IL-2-NAEi [126]
IBD	CRL4⊣Deptor⊣mTOR	Acceleration	MLN4924 [76]
GVHD	SAG	Acceleration	[96]
Obesity-related inflammation	UBE2M→TRIM21⊣VHL⊣HIF-1α→IL-1β	Acceleration	[129]
LPS-induced AKI	CRL1→NF-κB→TNF-α, IL-6, IL-1B	Acceleration	MLN4924 [130]
LPS-induced ALI	CRL5→TRAF6→NF-κB	Acceleration	[53]

SLE, systemic lupus erythematosus; TMPD, tetramethylpentadecane; RA, rheumatoid arthritis; IBD, inflammatory bowel disease; GVHD, graft-versus-host disease; LPS, lipopolysaccharide.

Taken together, it appears that neddylation modification plays a dual (pro- or anti-) role in the regulation of inflammatory diseases in a cell- and context-dependent manner. Caution is, therefore, required when targeting the neddylation pathway for the treatment of inflammatory diseases, where a thorough understanding of the underlying mechanism is essential.

## Conclusion and Future Perspectives

As a posttranslational modification, neddylation has been validated to regulate both innate and adaptive cellular immune responses. The defective neddylation could alter the characteristics and functions of immune cells, thereby contributing to the pathogenesis of diverse immune-related diseases, including cancer, infectious diseases, AIDs, and auto-inflammatory disorders. Some future perspectives are proposed below to broaden our mechanistic understanding and potential therapeutic applications, resulting from these exciting research fields of neddylation and immunology (Fig. 5).

First, how to improve the effectiveness of MLN4924 in cancer treatment remains elusive. Neddylation is often overactivated during tumorigenesis, and neddylation inhibition induces various types of tumor cell death as well suppresses the functions of immune cells within the TME, of which some are pro- and the others are anti-tumorigenesis. The net outcome of neddylation inhibition needs to be precisely defined in a particular cellular context, before a complementary immunotherapy should be applied as a combinational therapy.

Second, can MLN4924 or other neddylation inhibitors be applied in the treatment of inflammatory diseases? Existing studies have shown the dual role of neddylation in regulation of inflammatory diseases, yet the precise molecular mechanism underlying these opposite effects remains unclear and needs to be thoroughly elucidated before it becomes practicable.

Third, how can we broaden the potential application of neddylation inhibitors in the treatment of human cancers or inflammatory diseases? MLN4924 is an E1 inhibitor with associated side effects and off-target effects [11]. Discovery and development of small molecular inhibitors of neddylation E2s or E3s or specific types of CRL, which are currently undergoing [131–134], will offer better opportunities.
